# Animal Protein versus Plant Protein in Supporting Lean Mass and Muscle Strength: A Systematic Review and Meta-Analysis of Randomized Controlled Trials

**DOI:** 10.3390/nu13020661

**Published:** 2021-02-18

**Authors:** Meng Thiam Lim, Bernice Jiaqi Pan, Darel Wee Kiat Toh, Clarinda Nataria Sutanto, Jung Eun Kim

**Affiliations:** Department of Food Science & Technology, National University of Singapore, 3 Science Drive 3, Singapore 117543, Singapore; e0427298@u.nus.edu (M.T.L.); e0384141@u.nus.edu (B.J.P.); dareltoh@u.nus.edu (D.W.K.T.); e0254848@u.nus.edu (C.N.S.)

**Keywords:** body composition, muscle mass, muscular strength, protein source

## Abstract

Although animal protein is usually considered to be a more potent stimulator of muscle protein synthesis than plant protein, the effect of protein source on lean mass and muscle strength needs to be systematically reviewed. This study aimed to examine potential differences in the effect of animal vs. plant protein on lean mass and muscle strength, and the possible influence of resistance exercise training (RET) and age. The following databases were searched: PubMed, Embase, Scopus and CINAHL Plus with Full Text, and 3081 articles were screened. A total of 18 articles were selected for systematic review, of which, 16 were used for meta-analysis. Total protein intakes were generally above the recommended dietary allowance at the baseline and end of intervention. Results from the meta-analyses demonstrated that protein source did not affect changes in absolute lean mass or muscle strength. However, there was a favoring effect of animal protein on percent lean mass. RET had no influence on the results, while younger adults (<50 years) were found to gain absolute and percent lean mass with animal protein intake (weighted mean difference (WMD), 0.41 kg; 95% confidence interval (CI) 0.08 to 0.74; WMD 0.50%; 95% CI 0.00 to 1.01). Collectively, animal protein tends to be more beneficial for lean mass than plant protein, especially in younger adults.

## 1. Introduction

Skeletal muscle is known to support physical stability and enable movement. It also has important metabolic functions, such as supplying amino acids during the post-absorptive state for tissue building and maintenance [1] and serving as a site for glucose uptake and storage [2]. Loss of muscle has detrimental consequences; low muscle mass has been associated with increased morbidity, poorer quality of life and higher mortality [3]. Similarly, low muscle strength was shown to be a significant and independent predictor of mortality risk [4,5].

A decline in muscle mass and strength is usually observed with age across different populations [6,7,8]. Muscle protein anabolism in older adults may be negatively affected by inadequate nutritional intake or impaired response to nutrients and hormones [9]. This age-related loss of muscle mass and strength is termed sarcopenia, now recognized as a “muscle disease” [10]. The development and progression of sarcopenia is influenced by peak muscle mass and strength attained in early adulthood, as well as their preservation later in life [11]. Preventing and treating low muscle mass or sarcopenia will not only lead to potential clinical benefits, but may also result in cost savings for the healthcare system [3,12].

Maintenance of muscle mass is a dynamic balance between muscle protein synthesis (MPS) and muscle protein breakdown (MPB). Muscle gain occurs only when MPS exceeds protein degradation (i.e., positive net protein balance). MPS is increased after resistance exercise, but protein balance remains negative as the rate of MPB is also elevated [13,14]. To achieve a positive net balance, ingestion of dietary protein is required [14]. Animal protein, with its higher protein quality, is usually considered to be superior to plant protein for building muscle mass [15,16,17]. On the other hand, healthcare professionals have encouraged the replacement of animal protein, particularly red meat, with plant protein to help decrease the risk of cardiovascular diseases [18]. Plant protein utilization is also promoted to reduce harm to the environment by decreasing the demand for animal protein, since animal farming tends to be more resource intensive with higher greenhouse gas emissions [19].

The effects of animal protein vs. plant protein on muscle mass and strength have been examined in a few systematic reviews, but there are research gaps. One publication concluded that a higher amount of plant protein is needed to achieve muscle growth similar to animal protein [20]. However, the review included trials which only studied acute changes in muscle protein turnover. This may not be appropriate since muscle hypertrophy is a result of long-term change in net protein balance [21]. Furthermore, the sole focus of that review was on adults below 40 years of age. It is important to understand the impact among older adults, since a substantial decline in muscle mass and muscle strength is known to occur after the age of about 50 years [10,22]. A recent meta-analysis concluded that soy protein resulted in similar muscle mass and strength gains as animal protein [23], but the authors did not investigate the use of other plant proteins or stratify their analyses according to age. In addition, both publications only reviewed studies in which subjects underwent resistance exercise training (RET). Potential differences in the effects of animal protein and plant protein among adults who do not engage in RET are therefore not known. Hence, the aim of the present systematic review and meta-analysis of randomized controlled trials (RCTs) was to compare the non-acute effects of animal protein vs. plant protein on muscle accretion and strength among adults ≥19 years, with and without RET.

## 2. Materials and Methods

The protocol for this study is in accordance with the PRISMA (Preferred Reporting Items for Systematic Reviews and Meta-Analyses) guidelines [24]. The description of the PICOS (population, intervention, comparison, outcome and setting) criteria used to define the research question is presented in Table 1 below.

### 2.1. Search Strategy and Inclusion Criteria

A computerized search of the literature was performed independently by a primary reviewer (M.T.L.) and a secondary reviewer (B.J.P.) for all articles from inception to early January 2020 using four online databases: namely, PubMed, Embase, Scopus and CINAHL Plus with Full Text. The search strategy focused on combining the terms “protein” or “proteins” with types of animal protein, types of plant protein and muscle mass or muscle strength. Medical subject headings were used where possible, and no filters were applied. An updated search was performed in mid-June 2020. Details of the search strategy can be found in Supplementary Materials (Table S1).

A total of 3081 articles were retrieved and exported into EndNote X9 (Clarivate Analytics) for literature management. After the exclusion of duplicates (*n* = 1150), screening was conducted based on inclusion criteria determined a priori. Studies had to be human RCTs, with subjects having a mean age of ≥19 years and written in English. The study must have allocated subjects to an animal protein group and a plant protein group. If the supplement given consisted of a protein blend, i.e., a mix of animal protein and plant protein (regardless of proportion), the study would not be accepted. Other than studies which provided protein as a supplement, those that specifically compared the effects of diets higher in animal protein and plant protein were also considered for inclusion. In both cases, it is reasonable to assume that subjects would have consumed more animal protein and plant protein in their overall diet, based on the intervention group they were assigned to. In line with this notion, studies which provided a different quantity of protein for each intervention group were included as well.

Finally, studies which only examined muscle protein fractional synthetic rate or net protein balance, without tracking changes in muscle mass, percent muscle mass and/or muscle strength, were excluded. Methods of body composition assessment accepted for this review were dual-energy X-ray absorptiometry (DEXA) and air displacement plethysmography (ADP). DEXA has been recommended as the reference standard for measuring muscle mass [25]. Measurement of fat-free mass in adults using DEXA and ADP was found to have a strong correlation [26]. For strength, the outcomes of interest were one-repetition maximum (1-RM) bench press and squat, grip strength, as well as peak torque of leg/knee extension and flexion.

### 2.2. Article Selection

**Figure 1:**
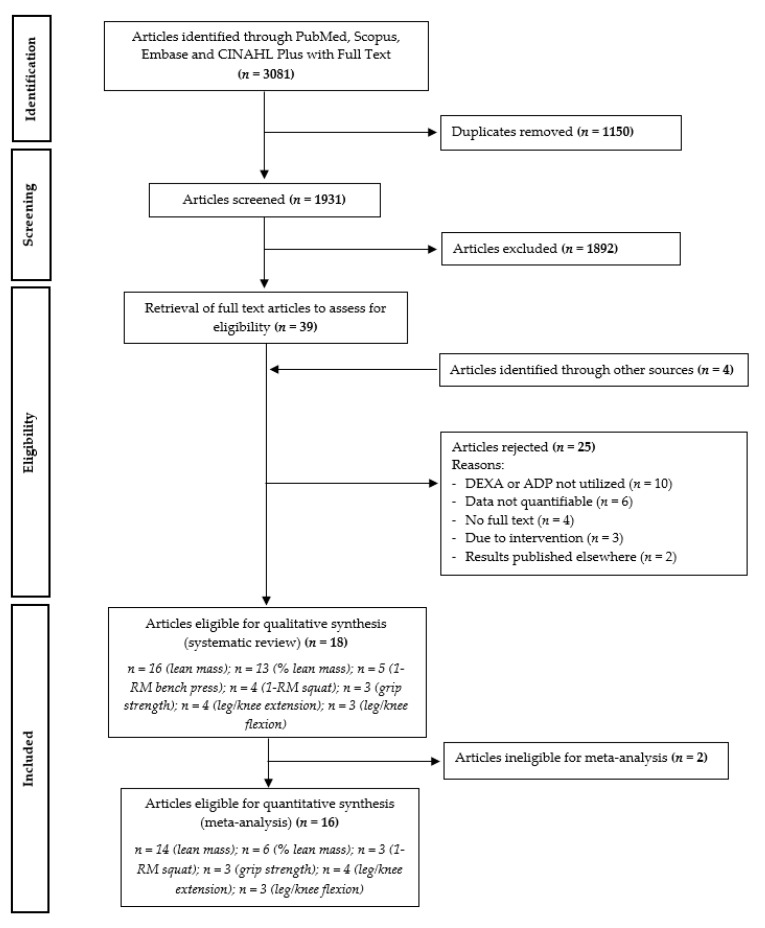
Preferred reporting items for systematic reviews and meta-analyses (PRISMA) flow chart of the literature selection process. 1-RM: one-repetition maximum.

The primary and secondary reviewers independently screened the titles and abstracts, and based on the pre-established criteria, eliminated 1892 articles. The full text for the remaining 39 articles were retrieved for further evaluation of inclusion eligibility; four original articles obtained from other sources were also added for assessment. Out of the total of 43 articles, 25 were subsequently rejected. Ten articles were omitted because DEXA or ADP was not found to be utilized for body composition assessment. Another six articles were excluded as they did not contain quantifiable values, i.e., results of interest were only presented in bar chart form. Four articles were rejected because no full text was available, while three articles were omitted as there was indication that the study intervention consisted of a protein blend. Two articles were removed since the relevant results were published in other eligible articles. Where necessary, attempts were made to contact corresponding authors to obtain data or seek clarification. Collectively, 18 articles were selected for this systematic review (Figure 1).

### 2.3. Data Extraction and Quality Assessment

The primary and secondary reviewers independently extracted details from the 18 selected articles onto an electronic form. The fields captured were the primary author’s last name; publication year; country; intervention period; study population; subjects’ gender, age, weight, height and body mass index; intervention specifics (type and amount of protein and intake protocol); description of usual diet; dietary assessment method; total protein consumed (g/kg/day, baseline and end-of-intervention) and inclusion of RET. Protein intake, if not reported, was estimated by dividing the mean total protein consumed with the corresponding mean body weight. Some articles only provided intake data which excluded protein supplementation. In such cases, end-of-intervention protein intake was estimated by adding the amount of supplemented protein to the final total protein consumed. The mean and standard deviation (SD) of the pre-intervention, post-intervention (final measurement) and change values of the outcome variables were extracted. Percent muscle mass, if not reported, was calculated by dividing muscle mass with the corresponding body weight at pre- and post-intervention, and change value was obtained by subtracting the final mean from the baseline mean. Relevant data provided by authors were added to the form, and where applicable, superseded existing values reported in or derived from the original articles.

Results from different intervention phases in a crossover trial were treated as if they were the respective groups in a parallel trial. If more than one variation of animal or plant protein was used in a study, the interventions were treated as independent trials and presented separately to account for within-study differences. Any data associated with the control group (i.e., non-protein intervention) were not captured since they were not relevant to our research question. When required, standard error (SE) values were converted to SD, while peak torque given in foot–pounds (ft lb) were standardized to Newton meters (Nm) by multiplying the value by 1.355818 [27]. Across the different articles, muscle mass was referred to using varying terminologies such as fat-free mass, lean body mass and lean tissue mass. For the purpose of this review, these terms were considered synonymous and “lean mass” is henceforth used consistently. By definition, bone is part of fat-free mass; bone is also sometimes included as part of lean body mass in the literature [28]. Nonetheless, studies that included bone mineral content within lean mass were still considered in our analyses, since bone only accounts for approximately 7% of fat-free mass (or lean body mass) [25]. Moreover, bone remodeling is a very slow process, lasting 4 to 6 months and may continue over a period of 2 years [29].

Risk of bias of the selected studies was evaluated using a modified version of the Cochrane risk of bias tool [30]. The primary and secondary reviewers independently assigned a subjective level of risk (low, high or unclear) for each study based on four domains: namely, random sequence generation, allocation concealment, blinding of participants and investigator and blinding of outcome assessors.

### 2.4. Calculations and Statistical Analyses

The reported and/or calculated change values from the different studies for each outcome were summarized and presented as a median. In addition, the range of change values were indicated for each outcome based on the minimum and maximum change values obtained from the studies.

In order to impute change SD for studies in which the value was missing, the correlation coefficient for a particular outcome was calculated based on at least one other study which was reported in considerable detail. The overall effect sizes of the outcomes were determined using weighted mean difference (WMD) of the change values between animal protein and plant protein groups, with 95% confidence intervals. Meta-analyses were performed using the metan function of the Stata/IC 13.0 software (StataCorp LP, College Station, TX, USA). A random-effects model was applied because effect sizes of the studies were expected to vary due to differences in the mix of subjects and interventions [31]. Sensitivity analysis was performed for each outcome based on the leave-one-out method to explore the potential effect of removing a single trial or pairwise comparison at a time.

Subgroup analyses were determined a priori to identify possible variations of observed effects in the overall analysis. Studies were categorized into those which provided RET as part of the protocol and those that did not, as well as younger (<50 years) and older (≥50 years) age groups.

## 3. Results

### 3.1. Characteristics of Selected Studies

Detailed characteristics of the selected studies are summarized in Table 2. All 18 studies were utilized for systematic review [32,33,34,35,36,37,38,39,40,41,42,43,44,45,46,47,48,49], while 16 studies [32,33,35,36,37,38,39,40,41,43,44,45,46,47,48,49] were eligible for meta-analysis. Of the 18 studies, 17 had a parallel design and one was a crossover study [45]. The duration of intervention ranged from 14 days to 2 years. In 11 studies, subjects participated in a RET program. Eight studies were conducted in subjects with a mean/median age of 50 years and older. Two studies involved trained subjects, while four studies recruited subjects with medical conditions (i.e., chronic kidney disease, hyperlipidemia, insulin resistance or metabolic syndrome).

The protein provided was used as a supplement in 15 studies. For animal protein, these encompassed whey (isolate, concentrate and hydrolysate), casein, milk protein (casein plus whey), dairy product and beef. Sources of plant protein were soy (isolate, concentrate and soy products), pea protein and rice protein isolate. In the remaining three studies, subjects were specifically assigned to diets which were higher in animal protein or plant protein [33,38,45]. Six studies utilized two variations of either animal or plant protein in the intervention, thus allowing for two pairwise comparisons from a single trial [33,38,40,43,44,49]. At baseline, subjects were generally consuming protein above the recommended dietary allowance (RDA) of 0.8 g/kg/day [50]. Subjects were estimated to have achieved a final protein intake of at least 1.0 g/kg/day; the highest total reported was 3.1 g/kg/day.

### 3.2. Quality of Selected Studies

Under selection bias, five and seven studies, respectively, provided sufficient information on randomization and allocation concealment. The remaining studies were judged to have unclear risk for these two domains. Blinding of participants may not always be feasible, especially if food was provided. In such cases, consideration will be made as to whether blinding of the investigator was carried out in order to evaluate the risk of performance bias. Eight RCTs were deemed as low risk for participant/investigator blinding, while six RCTs were judged likewise for blinding of outcome assessors (detection bias). The risk for the other studies was considered to be unclear. Overall, two RCTs were found to be at low risk across all four domains. Details of each study’s risk of bias assessment are available in Supplementary Materials (Table S2).

### 3.3. Results of Systematic Review

The summary of the published results on the impact of protein source on changes in lean mass and muscle strength is shown in Table 3. On the whole, consumption of both animal protein and plant protein demonstrated an increase in the median value of lean mass and strength outcomes. Animal protein presented greater gains for lean mass and percent lean mass compared to plant protein, while findings for strength outcomes were inconsistent.

### 3.4. Results of Meta-Analysis

**Figure 2:**
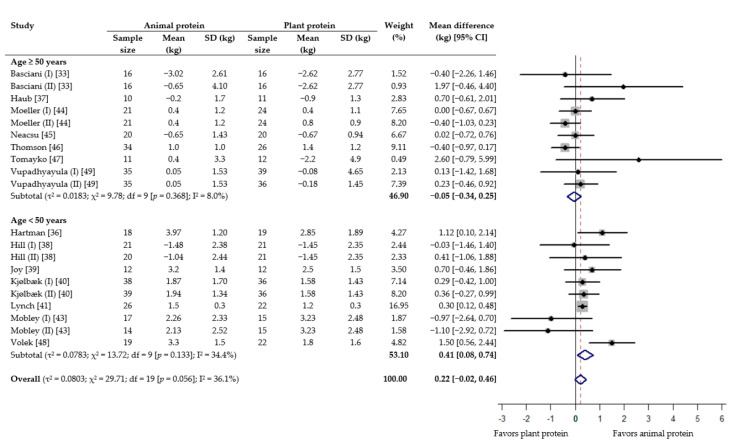
Effect of consuming animal protein compared to plant protein on changes in absolute lean mass (kg) based on age group. Data expressed as weighted mean differences with 95% CIs, using a random-effects model.

**Figure 3:**
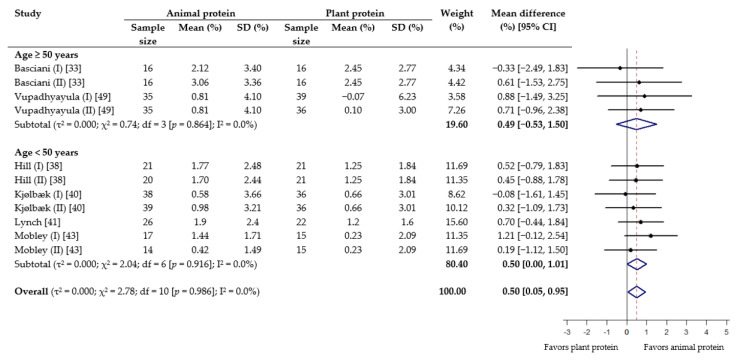
Effect of consuming animal protein compared to plant protein on changes in percent lean mass (%) based on age group. Data expressed as weighted mean differences with 95% CIs, using a random-effects model.

Meta-analysis revealed that although consuming animal protein provided a favorable effect on absolute lean mass compared to plant protein, the result was not statistically significant (WMD 0.22 kg; 95% CI −0.02 to 0.46) (Figure 2). On the other hand, animal protein intake was found to produce a statistically significant increase in percent lean mass (WMD 0.50%; 95% CI 0.05 to 0.95) (Figure 3). In the subgroup analysis based on age, while no difference was seen among older adults (≥50 years), there was a gain of 0.41 kg lean mass (95% CI, 0.08 to 0.74) and 0.50% lean mass (95% CI 0.00 to 1.01) with animal protein intake among subjects <50 years (Figure 2 and Figure 3). When analyzed according to RET, results showed no significant difference between the effect of protein source on absolute and percent lean mass, with or without RET (Figures S1 and S2).

**Figure 4:**
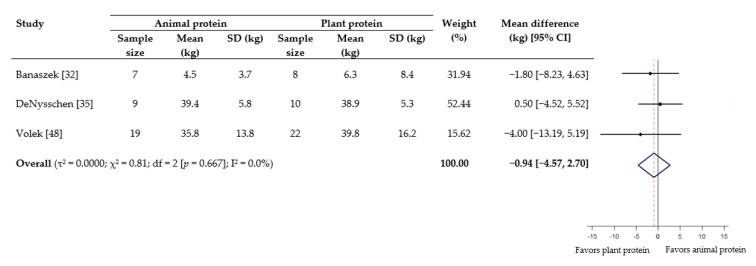
Effect of consuming animal protein compared to plant protein on changes in 1-RM squat (kg). Data expressed as weighted mean differences with 95% CIs, using a random-effects model. 1-RM: one-repetition maximum.

**Figure 5:**
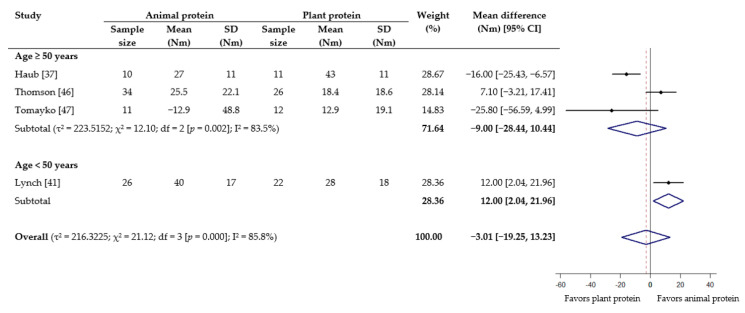
Effect of consuming animal protein compared to plant protein on changes in peak torque of leg/knee extension (Nm) based on age group. Data expressed as weighted mean differences with 95% CIs, using a random-effects model. Nm: Newton meter.

**Figure 6:**
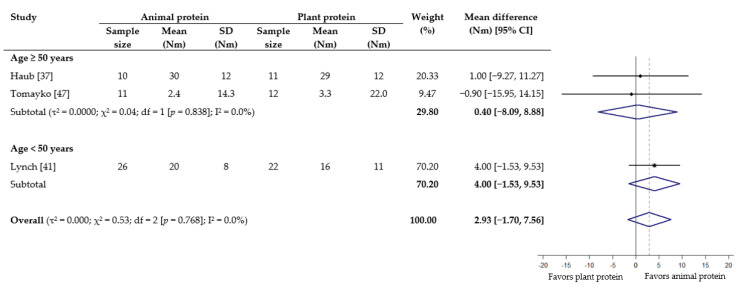
Effect of consuming animal protein compared to plant protein on changes in peak torque of leg/knee flexion (Nm) based on age group. Data expressed as weighted mean differences with 95% CIs, using a random-effects model. Nm: Newton meter.

As for muscle strength, meta-analyses showed no statistical difference in effect between animal protein and plant protein for 1-RM squat (WMD −0.94 kg; 95% CI −4.57 to 2.70) (Figure 4), grip strength (WMD −0.49 kg, 95% CI −1.28 to 0.30) (Figure S3), leg/knee extension (WMD −3.01 Nm; 95% CI −19.25 to 13.23) (Figure 5) and leg/knee flexion (WMD 2.93 Nm; 95% CI −1.70 to 7.56) (Figure 6). For the subgroup analyses based on age, a significant effect favoring animal protein was found in subjects <50 years for peak torque of leg/knee extension (WMD 12.00 Nm; 95% CI 2.04 to 21.96), although this was not seen for leg/knee flexion (Figure 5 and Figure 6). The subgroup analyses according to provision of RET did not demonstrate any significant difference between the effect of protein source on the measurements of muscle strength (Figures S3–S5).

Overall, there may be moderate heterogeneity across studies for absolute lean mass (I^2^ = 36.1%; *p*-value from Chi-squared test = 0.056), while heterogeneity was not present for percent lean mass (I^2^ = 0.0%; *p* = 0.986). Results for both lean mass outcomes were not stable to the leave-one-out sensitivity analysis (Table S3). For absolute lean mass, the impact of animal protein became significant when the study by Mobley et al. [43], Moeller et al. [44] or Thomson et al. [46] was removed from the analysis, resulting in higher WMD of between 0.25 kg and 0.29 kg. Percent lean mass was highly affected by four studies: i.e., Hill et al. [38], Lynch et al. [41], Mobley et al. [43] and Vupadhyayula et al. [49]. When either one of these studies was removed from the analysis, the impact of animal protein on percent lean mass was no longer significant.

In terms of muscle strength outcomes, there was no heterogeneity for the 1-RM squat, grip strength and peak torque of leg/knee flexion (I^2^ = 0.0% for all). However, considerable heterogeneity was found for peak torque of leg/knee extension (I^2^ = 85.8%; *p* = 0.000). The results reported for strength were all robust to sensitivity analysis (Table S4).

## 4. Discussion

Even though there is general consensus that animal protein is a potent stimulator of MPS, the effect of protein source on lean mass accretion over time and the potential influence of RET and age has not been systematically reviewed. Our qualitative assessment showed that both animal protein and plant protein supported an increase in absolute and percent lean mass, although a more substantial gain was observed with animal protein. Quantitatively, the meta-analysis revealed a favoring effect of animal protein specifically for percent lean mass. There was a significant gain in both absolute and percent lean mass with animal protein intake among adults <50 years, while RET did not influence the effect of protein source on changes in lean mass.

The positive impact of animal protein on percent lean mass could be attributed to its protein quality. Protein quality is dependent on the composition of amino acids as well as its ability to be digested, absorbed and utilized to meet the body’s needs [51]. Animal protein is deemed as “high quality” because it provides all the essential amino acids (EAAs) in sufficient quantities, and tends to be well digested [52]. EAAs are known to stimulate the mammalian target of rapamycin complex 1 (mTORC1) signaling pathway, triggering a rise in MPS [53,54]. Plant protein, including soy, is deficient in specific EAAs [15,52]. This relative lack of EAAs in plant protein may result in their amino acids being directed towards urea synthesis, instead of muscle building [15]. In addition, plant protein is generally less digestible than animal protein likely due to differences in protein structure, thus affecting their anabolic potential [17]. Since percent lean mass takes into account body weight, it is also plausible that subjects who consumed proportionally more animal protein experienced a greater loss or lesser gain in body weight (fat) over time. This is because the ingestion of animal protein may induce higher energy expenditure than plant protein, possibly due to its greater anabolic effect [52]. Increased percent lean mass has been shown to be associated with desirable health outcomes, such as lower risk of metabolic syndrome [55] and reduced mortality risk among middle-aged women [56].

Interestingly, absolute lean mass was not shown to be affected by protein source in our meta-analysis. Most subjects in the included studies were consuming a varied diet, comprising different protein foods. Hence, the finding that protein source did not differentially affect absolute lean mass may suggest that the proportion of animal protein and plant protein in a diverse diet do not influence the chronic response of muscle turnover, provided the total protein consumed is adequate. As stated before, subjects in the studies reviewed here achieved a total protein intake above the RDA, regardless of intervention group. Li et al. analyzed the diets of over 3200 community-dwelling adults and found no association between the ratio of animal-to-plant protein intake and the lean mass of participants, as measured by skeletal muscle index (SMI). There was however a significant relationship between total protein intake and SMI, where higher SMI was seen with protein intakes greater than the RDA [57]. Nonetheless, the discrepancy in results seen for the effect of animal protein on absolute and percent lean mass warrants further investigation. It should be noted that the number of studies used to assess the impact of protein source on absolute lean mass is different from that used for percent lean mass.

Morton et al. previously demonstrated in a systematic review that protein supplementation augmented gains in fat-free mass in response to RET, up to intakes of ~1.6 g/kg/day [58]. The authors however found no significant role for protein source (soy vs. whey) on changes in fat-free mass. In line with this, our subgroup analyses revealed that animal protein and plant protein did not differentially affect absolute and percent lean mass among subjects who performed RET. In the absence of RET, it has been shown that protein intakes greater than the RDA did not induce significant changes in lean mass over time [59]. We have further demonstrated here that without RET, protein source similarly had no influence on changes in absolute and percent lean mass. The revelation that both the quantity and quality of protein had no effect on lean mass, in the absence of RET, is perhaps not unexpected. MPS is known to switch off after a certain duration despite sustained amino acid availability, and RET is able to delay this “set-point” by up to and beyond 24 h [60]. In other words, the combination of protein intake and exercise is expected to be more anabolic than protein alone.

We found a significant gain in both absolute and percent lean mass with animal protein intake among adults <50 years, an effect not seen in older adults (≥50 years). Animal protein generally contains higher EAA content than plant protein [61], and evidence suggests that young muscles are more sensitive to the anabolic action of EAAs compared to aging muscles [62]. It is generally recognized that there is an attenuated response of MPS to the ingestion of protein which occurs with aging—a phenomenon known as “anabolic resistance” [63,64]. The etiology for this condition is not fully understood, but could be related to defects caused by declining physical activity, prolonged muscle disuse or chronic inflammation [65]. The cellular mechanisms may involve impaired activation of mTORC1 and downstream targets implicated in translation initiation, such as ribosomal protein S6 kinase (p70S6K) and eukaryotic initiation factor 4E binding protein 1 (4EBP1) [16]. While this age-associated reduction in MPS for older adults could be enhanced with greater doses of protein [62], the provision of more EAAs will not elevate MPS to the rate seen in younger adults [63]. Our finding appears to support the notion that protein intake may need to be enriched with other nutritional compounds, such as beta-hydroxy-beta-methylbutyrate (HMB) and vitamin D, to help maintain muscle mass among middle-aged and older adults [66].

Although animal protein was found to have resulted in a statistically significant gain in percent lean mass, as well as absolute and percent lean mass among younger adults (<50 years), the clinical significance of this increase is unclear. Based on the confidence interval of our results, the maximum gain in percent lean mass with animal protein intake was 0.95% overall. In a retrospective study conducted among Korean adults, the average percent lean mass of individuals with no metabolic syndrome was found to be approximately 1% higher than those with the condition [55]—this might be suggestive of the practical importance of our data. Regardless, as there is currently no consensus on what represents a minimal clinically important difference in lean mass [67], healthcare professionals should exercise appropriate clinical judgement in the interpretation of our findings.

The effect of protein source on muscle strength was found to be inconsistent across different outcome measures, based on the current qualitative assessment—this is reflective of results from observational studies [68,69]. Nevertheless, our meta-analyses revealed that protein source did not affect changes in strength outcomes. Similarly, no differences were found in subgroup analyses based on RET. This echoes findings by Messina et al., who demonstrated that both soy and animal protein (whey, beef and dairy) produced significant increases in strength (1-RM bench press and 1-RM squat) in response to RET, with no difference between protein groups [23]. Indeed, RET has been shown to be a far more potent stimulus for increasing muscle strength than protein supplementation [58]. It is therefore not surprising to find that without RET, protein source also did not differentially affect strength. As for subgroup analyses based on age, a significant effect favoring animal protein was seen in subjects <50 years for peak torque of leg/knee extension only. Although animal protein was found to benefit lean mass in younger adults in this study, lean mass gain may not necessarily translate to strength improvements. The link between growth in lean mass and changes in muscle strength is still a matter of debate [70,71]. Overall, there is no difference in effect between animal protein and plant protein on strength outcomes, with or without RET; the influence of age is not clear. It should be noted that the small number of studies used in these meta-analyses has limited our ability to draw any definitive conclusions.

The present work makes a unique contribution with its wide inclusion criteria that were not restricted to particular protein types or narrowly-defined participant characteristics. This has allowed us to conduct a comprehensive overview of the topic and increase the external validity and generalizability of our results. In addition, the omission of acute trials in this systematic review more accurately represents the accretion of lean mass that occurs over time. Nonetheless, our systematic review also has several limitations. Considering the variability in subjects and interventions of the included studies, there could have been a masking effect on the pooled estimates. Although subgroup analyses were conducted to uncover potential differences, findings were limited to the influence of age and RET. Moreover, findings for both absolute and percent lean mass were not stable to leave-one-out sensitivity analyses, hence our results need to be interpreted with caution. Although this review aimed to compare the effects between animal protein and plant protein, it should be noted that the source of animal protein in most studies was derived from dairy, while for plant protein a majority of the studies utilized some form of soy protein.

Another potential limitation is that we have assumed subjects randomized to the animal protein group and plant protein group have consumed a diet which was higher in animal protein and plant protein, respectively. It is however not possible to confirm this assumption for studies which provided protein as a supplement, since no details on background dietary pattern or intake were reported. For example, an individual assigned to receive a plant protein supplement could be eating a lot of animal-based foods, resulting in higher animal protein intake overall. It is imperative for future studies that aim to investigate the effect of protein source on lean mass and strength to include data on the subjects’ background diet, since the lack of such information may compromise the validity of the study’s results.

## 5. Conclusions

In summary, this systematic review and meta-analysis have found that animal protein tends to have a more favorable effect on lean mass compared to plant protein, and the benefit appears more pronounced in younger adults. On the other hand, protein source is not likely to have an impact on muscle strength.

## Figures and Tables

**Table 1:** Population, intervention, comparison, outcome and setting (PICOS) criteria used to define the research question.

Parameter	Description
Population	Adults with mean age ≥19 years
Intervention	Consumption of animal protein, as food or supplement
Comparator	Consumption of plant protein, as food or supplement
Outcome	Change in muscle mass and/or muscle strength
Study design	Randomized controlled trials
Research question	Are there differences in the effect of animal protein and plant protein on muscle mass and strength in adults?

**Table 2:** Summary of selected studies for systematic review and meta-analysis.

Author, Year and Country	Duration of Intervention	Subjects	Intervention (per day)	n ^†^/Gender	Age (years)	Baseline Weight (kg)	Total Protein Intake ^^^		RET	Reported Outcomes ^#^
							Baseline	Final		
Banaszek et al. (2019), USA [32]	8 weeks	Healthy, trained adults	Whey protein: 48.8 g Pea protein: 49 g	4M/3F 4M/4F	M:38.6 ± 12.7; F:38.9 ± 10.9	83.9 ± 18.9 78.4 ± 11.6	NA	1.8 ± 0.3 ^a^ 1.7 ± 0.4 ^a^	Yes	1-RM squat
Basciani et al. (2020), Italy [33]	~6 weeks (45 days)	Obese, insulin-resistant, untrained adults	Whey protein ^b^ Meat, fish and eggs ^b^ Plant protein ^b^	16 16 16	56.2 ± 6.1	102.02 ± 12.04 98.36 ± 14.49 102.10 ± 12.36	NA	~1.0	No	Total lean (DEXA); grip strength
Candow et al. (2006), Canada [34]	6 weeks	Healthy, untrained adults	Whey protein: ~83 g Soy protein: ~86 g	6F/3M 6F/3M	24.0 ± 6 22.5 ± 6	69.3 ± 12 71.8 ± 15	1.6 1.8	3.1 3.0	Yes	Lean tissue mass (DEXA); 1-RM bench press; 1-RM squat
DeNysschen et al. (2009), USA [35]	12 weeks	Overweight, untrained men with hypercholesterolemia	Whey protein: 26.6 g Soy protein: 25.8 g	9M 10M	21–50	90 ± 13.2 92.9 ± 7.9	1.0 ± 1.5 0.92 ± 0.9	1.2 ± 0.9 1.1 ± 0.9	Yes	1-RM bench press; 1-RM squat
Hartman et al. (2007), Canada [36]	12 weeks	Healthy, untrained young men	Milk: 35 g Soy beverage: 35 g	18M 19M	18–30	78.8 ± 10.6 83.3 ± 17.9	1.4 ± 0.4 1.2 ± 0.4	1.8 ± 0.8 1.6 ± 0.4	Yes	Fat- and bone-free mass (DEXA)
Haub et al. (2002), USA [37]	12 weeks	Healthy, untrained older men	Beef: ~54 g TVP: ~54 g	10M 11M	63 ± 3 67 ± 6	89.5 ± 8.7 89.1 ± 6.3	1.00 ± 0.2 1.06 ± 0.1	1.03 ± 0.3 1.15 ± 0.1	Yes	Fat-free mass (ADP); leg extension and flexion
Hill et al. (2015), USA [38]	6 months	Overweight/obese untrained adults with metabolic syndrome	Animal foods: 102.2 g ^c^ Animal foods: 63.7 g ^c^ Plant foods: 64.3 g ^c^	10M/11F 9M/10F 9M/12F	46.4 ± 8.5 46.2 ± 9.4 45.3 ± 6.7	104.8 ± 17.7 101.8 ± 15.6 102.1 ± 15.5	~0.9 ^d^ ~0.9 ^d^ ~0.9 ^d^	~1.5 ^e^ ~1.1 ^e^ ~1.0 ^e^	No	Body lean mass (DEXA)
Joy et al. (2013), USA [39]	8 weeks	Healthy, trained young men	Whey protein isolate: 48 g Rice protein isolate: 48 g	12M 12M	21.3 ± 1.9	76.08 ± 5.6	NA	NA	Yes	Lean body mass (DEXA); 1-RM bench press
Kjølbæk et al. (2017), Denmark [40]	24 weeks	Healthy, overweight/obese, untrained adults	Whey protein + calcium: 45 g Whey protein: 45 g Soy protein isolate: 45 g	7M/31F 7M/32F 8M/28F	42.7 ± 10.5 42.2 ± 9.32 42.4 ± 9.65	96.2 ± 14.5 95.8 ± 13.5 96.9 ± 13.2	1.00 ± 0.29 1.02 ± 0.27 1.00 ± 0.26	1.58 ± 0.29 1.66 ± 0.36 1.57 ± 0.36	No	Lean body mass (DEXA)
Lynch et al. (2020), USA [41]	12 weeks	Healthy, untrained adults	Whey protein isolate: 19 g Soy protein isolate: 26 g	10M/16F 7M/15F	18–35	66.9 ± 10.1 65.5 ± 13.3	1.4 1.2	~1.6 ~1.8	Yes	Lean body mass (DEXA); leg extension and flexion
Maltais et al. (2016), Canada [42]	16 weeks	Sarcopenic, untrained older men	Milk: 13.53 g Soy beverage + EAA powder: 12 g	8M 8M	68 ± 5.6 64 ± 4.8	76.7 ± 9.0 80.5 ± 13.5	1.04 1.26	~1.13 ~1.36	Yes	Lean body mass (DEXA); 1-RM bench press
Mobley et al. (2017), USA [43]	12 weeks	Healthy, untrained young men	Whey protein concentrate: 52.6 g Whey protein hydrolysate: 50.8 g Soy protein concentrate: 78.4 g	17M 14M 15M	21 ± 4.1 21 ± 3.7 21 ± 3.9	81 ± 12.4 79 ± 11.2 81 ± 11.6	1.1 ± 0.4 1.2 ± 0.4 1.1 ± 0.4	1.8 ± 0.41 1.9 ± 0.37 2.1 ± 0.39	Yes	Lean body mass (DEXA)
Moeller et al. (2003), USA [44]	24 weeks	Healthy, perimenopausal, untrained women	Whey protein: 40 g Soy protein (isoflavone-poor): 40 g Soy protein (isoflavone-rich): 40 g	21F 24F 24F	49.4 ^f^ 50.9 ^f^ 50.2 ^f^	64.6 ± 8.9 64.5 ± 8.1 66.8 ± 10.2	~1.1 ~1.1 ~1.0	Mean intake +27 g among all subjects	No	Bone-free lean mass (DEXA)
Neacsu et al. (2005), UK [45]	2 weeks (crossover)	Healthy, overweight/obese, untrained men	Meat (chicken and beef) ^g^ Soy foods and TVP ^g^	20M	51 ± 11.4	109.6 ± 17.2	~1.1 ^h^ ~1.1 ^h^	~1.5 ^e^ ~1.5 ^e^	No	Fat-free mass (ADP)
Thomson et al. (2016), Australia [46]	12 weeks	Healthy, untrained adults	Dairy shake: 27 g ^i^ Soy shake: 27 g ^i^	34 26	61.3 ± 6.9 61.7 ± 8.3	77.7 ± 15.6 75.8 ± 12.6	NA	1.42 ± 0.14 ^a^ 1.45 ± 0.14 ^a^	Yes	Total body lean mass (DEXA); grip strength; knee extension
Tomayko et al. (2015), USA [47]	6 months	Adults on maintenance hemodialysis	Whey protein: 27 g Soy protein: 27 g	7M/4F 7M/5F	57.0 ± 4.8 52.5 ± 4.3	89.8 ± 24.5 91.9 ± 19.4	NA	NA	No	Whole body lean mass (DEXA); leg extension and flexion
Volek et al. (2013), USA [48]	9 months	Healthy, untrained adults	Whey protein concentrate: 21.6 g Soy protein isolate: 20.0 g	13M/6F 11M/11F	22.8 ± 3.7 24.0 ± 2.9	74.1 ± 15.7 72.0 ± 8.4	1.27 ± 0.41 1.27 ± 0.45	1.39 ± 0.18 1.35 ± 0.13	Yes	Lean body mass (DEXA); 1-RM bench press; 1-RM squat
Vupadhyayula et al. (2009), USA [49]	24 months	Healthy, postmenopausal, untrained women	Casein + whey: 25 g Soy protein isolate + isoflavone: 25 g Soy protein isolate: 25 g	52F 57F 48F	63.9 ± 4.3 63.8 ± 4.6 63.6 ± 4.5	69.6 ± 11.5 70.4 ± 12.0 71.4 ± 10.7	0.93 ± 0.21 0.97 ± 0.25 0.88 ± 0.26	1.34 ± 0.26 1.17 ± 0.30 1.07 ± 0.30	No	Lean body mass (DEXA); grip strength

All values are mean ± SD, unless otherwise stated. Abbreviations: 1-RM, one-repetition maximum; ADP, air displacement plethysmography; DEXA, dual-energy X-ray absorptiometry; EAA, essential amino acid; NA, not available; RET: resistance exercise training; TVP, textured vegetable protein (soy); ^†^ Based on subjects with reportable results, and may not reflect initial number recruited; ^^^ g/kg/day, unless otherwise stated; **^#^** Only outcomes utilized in the current review are shown; ^a^ Average intake during study period. Baseline and final intake values were not reported; ^b^ Subjects in each group were given 90 g protein as part of a very-low-calorie ketogenic diet. Plant protein was derived from soy, green peas or cereals. Quantity for each protein source was not specified; ^c^ Subjects were assigned to one of three diets: a diet in which plant protein (pulses, grains, soy, nuts and seeds) contributed two-thirds of total protein, a diet in which animal protein (lean beef, chicken, tuna, eggs and dairy) contributed two-thirds of total protein, and a higher protein diet where animal protein contributed two-thirds of total protein; ^d^ Calculated based on protein content of a 2-week controlled feeding diet, provided prior to randomization. Intake data were not reported; ^e^ Calculated based on protein content of the intervention diet. Intake data were not reported; ^f^ Median values. Median age of all subjects was 50.6 years; ^g^ Total protein provided in the meat-based diet and soy-based diet was 154.74 g and 153.03 g, respectively. Quantity for each protein source was not specified; ^h^ Calculated based on protein content of a 3-day maintenance diet, provided prior to randomization. Intake data were not reported; ^i^ Dairy shake consisted of reduced-fat milk, no-fat yoghurt and vanilla milk mix syrup. Soy shake was made from reduced-fat soy milk, soy yoghurt, soy protein powder and maltodextrin.

**Table 3:** Summary of changes in lean mass and strength from baseline levels after the consumption of animal protein compared to plant protein *.

Outcome	Protein Source			
	Animal Protein		Plant Protein	
	Median	Range	Median	Range
Lean mass (kg)	1.25	−3.02–3.97	0.80	−2.62–3.2
Percent lean mass (%)	1.50	−0.6–3.06	0.32	−3.3–2.9
1-RM bench press (kg)	9.00	7.06–20.1	12.75	7.6–18.2
1-RM squat (kg)	31.25	4.5–39.4	31.30	6.3–39.8
Grip strength (kg)	1.20	−1.59–1.98	0.09	−0.86–1.6
Leg/knee extension (Nm)	26.25	−12.9–40	23.20	12.9–43
Leg/knee flexion (Nm)	20.00	2.4–30	16.00	3.3–29

1-RM: one-repetition maximum; Nm: Newton meter * Summary of reported and/or calculated change values for each outcome, presented as the median and range (min–max) of change values. Median standardized to two decimal places. Change values derived from the following number of studies: lean mass (*n* = 16); percent lean mass (*n* = 13); 1-RM bench press (*n* = 5); 1-RM squat (*n* = 4); grip strength (*n* = 3); leg/knee extension (*n* = 4); leg/knee flexion (*n* = 3). For the study by Maltais et al., only data for animal protein were used in qualitative assessment. Data for plant protein were omitted due to the addition of essential amino acid powder.

## Supplementary Materials

The following are available online at https://www.mdpi.com/2072-6643/13/2/661/s1, Figure S1: Effect of consuming animal protein compared to plant protein on changes in absolute lean mass (kg), with and without resistance exercise training (RET). Data expressed as weighted mean differences with 95% CIs, using a random-effects model. Figure S2: Effect of consuming animal protein compared to plant protein on changes in percent lean mass (%), with and without resistance exercise training (RET). Data expressed as weighted mean differences with 95% CIs, using a random-effects model. Figure S3: Effect of consuming animal protein compared to plant protein on changes in grip strength (kg), with and without resistance exercise training (RET). Data expressed as weighted mean differences with 95% CIs, using a random-effects model. Figure S4: Effect of consuming animal protein compared to plant protein on changes in peak torque of leg/knee extension (Nm), with and without resistance exercise training (RET). Data expressed as weighted mean differences with 95% CIs, using a random-effects model. Figure S5: Effect of consuming animal protein compared to plant protein on changes in peak torque of leg/knee flexion (Nm), with and without resistance exercise training (RET). Data expressed as weighted mean differences with 95% CIs, using a random-effects model. Table S1: Search strategy for a systematic review and meta-analysis assessing the effects of animal protein versus plant protein on supporting muscle mass and strength in adults. Table S2: Risk of bias assessment of included studies. Table S3: Sensitivity analysis for lean mass outcomes following the removal of single groups or randomized controlled trials to assess the robustness of meta-analyses results. Table S4: Sensitivity analysis for strength outcomes following the removal of single groups or randomized controlled trials to assess the robustness of meta-analyses results.

## Abbreviations

1-RM	One repetition maximum
ADP	Air displacement plethysmography
DXA	Dual-energy X-ray absorptiometry
EAA	Essential Amino Acid
FMM	Fat-free mass
MPB	Muscle protein breakdown
MPS	Muscle protein synthesis
mTORC1	Mammalian target of rapamycin complex 1
RCT	Randomized controlled trial
RDA	Recommended dietary allowance
RET	Resistance exercise training
SMI	Skeletal muscle index
