# Supplementary material for: Animal Protein versus Plant Protein in Supporting Lean Mass and Muscle Strength: A Systematic Review and Meta-Analysis of Randomized Controlled Trials
Source: Nutrients. 2021 Feb 18;13(2):661. doi: 10.3390/nu13020661 (PMC7926405; doi:10.3390/nu13020661)
Supplement: Supplementary file 1 [file nutrients-13-00661-s001.zip › nutrients-1072651-supplementary.pdf]

Table S1. Search strategy for a systematic review and meta-analysis assessing the effects of animal protein versus plant protein on supporting muscle mass and strength in adults.

Database	Query	Search Results
PubMed	(((((protein[Title/Abstract]) OR proteins[Title/Abstract])) AND (((((((((((((meat proteins[MeSH Terms]) OR milk proteins[MeSH Terms]) OR egg proteins, dietary[MeSH Terms]) OR meat[Title/Abstract]) OR meats[Title/Abstract]) OR poultry[Title/Abstract]) OR fish[Title/Abstract]) OR fishes[Title/Abstract]) OR egg[Title/Abstract]) OR eggs[Title/Abstract]) OR milk[Title/Abstract]) OR dairy[Title/Abstract]) OR whey[Title/Abstract]) OR casein[Title/Abstract])) AND ((((((((((plant proteins, dietary[MeSH Terms]) OR plant[Title/Abstract]) OR plants[Title/Abstract]) OR legume[Title/Abstract]) OR legumes[Title/Abstract]) OR soy[Title/Abstract]) OR pea[Title/Abstract]) OR peas[Title/Abstract]) OR bean[Title/Abstract]) OR beans[Title/Abstract])) AND ((((((((((((muscle, skeletal[MeSH Terms]) OR muscle strength[MeSH Terms]) OR body composition[MeSH Terms]) OR muscle[Title/Abstract]) OR "muscle mass"[Title/Abstract]) OR "lean mass"[Title/Abstract]) OR "lean body mass"[Title/Abstract]) OR "fat-free mass"[Title/Abstract]) OR "fat-free body mass"[Title/Abstract]) OR "body composition"[Title/Abstract]) OR "muscle strength"[Title/Abstract]) OR "muscular strength"[Title/Abstract])	509
Embase	(protein:ab,ti OR proteins:ab,ti) AND ('animal protein'/exp OR 'egg protein'/exp OR 'milk protein'/exp OR meat:ab,ti OR meats:ab,ti OR poultry:ab,ti OR fish:ab,ti OR fishes:ab,ti OR egg:ab,ti OR eggs:ab,ti OR dairy:ab,ti OR milk:ab,ti OR whey:ab,ti OR casein:ab,ti) AND ('plant protein'/exp OR plant:ab,ti OR plants:ab,ti OR soy:ab,ti OR legume:ab,ti OR legumes:ab,ti OR pea:ab,ti OR peas:ab,ti OR bean:ab,ti OR beans:ab,ti) AND ('muscle mass'/exp OR 'muscle strength'/exp OR 'body composition'/exp OR 'body composition':ab,ti OR muscle:ab,ti OR 'muscle mass':ab,ti OR 'lean mass':ab,ti OR 'lean body mass':ab,ti OR 'fat-free mass':ab,ti OR 'fat-free body mass':ab,ti OR 'muscle strength':ab,ti OR 'muscular strength':ab,ti)	694
Scopus	(TITLE-ABS-KEY (protein OR proteins) AND TITLE-ABS-KEY (meat OR meats OR poultry OR fish OR fishes OR egg OR eggs OR dairy OR milk OR whey OR casein) AND TITLE-ABS-KEY (plant OR plants OR soy OR legume OR legumes OR pea OR peas OR bean OR beans) AND TITLE-ABS-KEY ("body composition" OR muscle OR "muscle mass" OR "lean mass" OR "lean body mass" OR "fat-free mass" OR "fat-free body mass" OR "muscle strength" OR "muscular strength"))	1659
CINAHL Plus with Full Text	protein OR proteins AND (MH "Milk Proteins+") OR (meat OR meats OR poultry OR fish OR fishes OR egg OR eggs OR dairy OR milk OR whey OR casein) AND (MH "Vegetable Proteins+") OR (plant OR plants OR soy OR legume OR legumes OR pea OR peas OR bean OR beans) AND ((MH "Muscle, Skeletal+") OR (MH "Muscle Strength+") OR (MH "Body Composition+")) OR ("body composition" OR muscle OR "muscle mass" OR "lean mass" OR "lean body mass" OR "fat-free mass" OR "fat-free body mass" OR "muscle strength" OR "muscular strength")	219
	**TOTAL**	**3081**

Table S2. Risk of bias assessment of included studies.

Author	Selection Bias		Performance Bias	Detection Bias
	Random Sequence Generation	Allocation Concealment	Blinding of Participants and Investigator	Blinding of Outcome Assessors
Banaszek et al. [32]	Unclear	Unclear	Unclear	Unclear
Basciani et al. [33]	Low risk	Unclear	Unclear	Unclear
Candow et al. [34]	Unclear	Low risk	Low risk	Unclear
DeNysschen et al. [35]	Unclear	Unclear	Low risk	Unclear
Hartman et al. [36]	Unclear	Unclear	Unclear	Unclear
Haub et al. [37]	Unclear	Unclear	Unclear	Unclear
Hill et al. [38]	Low risk	Unclear	Low risk	Low risk
Joy et al. [39]	Unclear	Unclear	Unclear	Unclear
Kjølbæk et al. [40]	Low risk	Low risk	Low risk	Low risk
Lynch et al. [41]	Unclear	Unclear	Unclear	Low risk
Maltais et al. [42]	Unclear	Low risk	Low risk	Unclear
Mobley et al. [43]	Low risk	Low risk	Low risk	Low risk
Moeller et al. [44]	Unclear	Unclear	Unclear	Unclear
Neacsu et al. [45]	Unclear	Unclear	Unclear	Unclear
Thomson et al. [46]	Unclear	Unclear	Unclear	Low risk
Tomayko et al. [47]	Unclear	Low risk	Low risk	Low risk
Volek et al. [48]	Unclear	Low risk	Unclear	Unclear
Vupadhyayula et al. [49]	Low risk	Low risk	Low risk	Unclear

Table S3. Sensitivity analysis for lean mass outcomes following the removal of single groups or randomized controlled trials to assess the robustness of meta-analyses results.

Group Excluded	Mean difference	[95% CI]	I^2 (%)	X^2	P for χ^2
<u>**Lean Mass (kg)**</u>					
None	0.215	[−0.024, 0.455]	36.1	29.71	0.056
Basciani et al. (I) [33]	0.226	[−0.019, 0.471]	38.5	29.26	0.045
Basciani et al. (II) [33]	0.198	[−0.038, 0.435]	35.2	27.76	0.066
Basciani et al. (I and II) [33]	0.208	[−0.034, 0.450]	37.8	27.31	0.054
Hartman et al. [36]	0.174	[−0.063, 0.411]	32.8	26.80	0.083
Haub et al. [37]	0.202	[−0.045, 0.449]	38.4	29.24	0.046
Hill et al. (I) [38]	0.223	[−0.025, 0.471]	39.1	29.58	0.042
Hill et al. (II) [38]	0.212	[−0.036, 0.461]	39.3	29.66	0.041
Hill et al. (I and II) [38]	0.220	[−0.037, 0.477]	42.4	29.53	0.030
Joy et al. [39]	0.199	[−0.049, 0.446]	38.1	29.10	0.047
Kjølbæk et al. (I) [40]	0.212	[−0.046, 0.471]	39.4	29.69	0.041
Kjølbæk et al. (II) [40]	0.206	[−0.055, 0.466]	39.1	29.57	0.042
Kjølbæk et al. (I and II) [40]	0.202	[−0.080, 0.484]	42.5	29.54	0.030
Lynch et al. [41]	0.213	[−0.078, 0.504]	37.2	28.65	0.053
Mobley et al. (I) [43]	0.236	[−0.001, 0.474]	35.0	27.67	0.067
Mobley et al. (II) [43]	0.235	[−0.002, 0.472]	34.8	27.63	0.068
Mobley et al. (I and II) [43]	0.254	[0.020, 0.488]	33.5	25.56	0.083
Moeller et al. (I) [44]	0.236	[−0.022, 0.493]	38.3	29.19	0.046
Moeller et al. (II) [44]	0.267	[0.029, 0.505]	29.4	25.51	0.111
Moeller et al. (I and II) [44]	0.293	[0.038, 0.549]	31.6	24.84	0.098
Neacsu et al. [45]	0.232	[−0.024, 0.487]	38.7	29.37	0.044
Thomson et al. [46]	0.273	[0.040, 0.507]	26.7	24.54	0.138
Tomayko et al. [47]	0.203	[−0.033, 0.439]	35.4	27.85	0.064

Volek et al. [48]	0.154	[−0.057, 0.364]	20.6	22.68	0.203
Vupadhyayula et al. (I) [49]	0.219	[−0.029, 0.467]	39.4	29.69	0.041
Vupadhyayula et al. (II) [49]	0.217	[−0.042, 0.476]	39.4	29.71	0.040
Vupadhyayula et al. (I and II) [49]	0.221	[−0.047, 0.490]	42.7	29.69	0.029
**Percent lean mass (%)**					
None	0.502	[0.053, 0.951]	0.0	2.78	0.986
Basciani et al. (I) [33]	0.539	[0.080, 0.998]	0.0	2.19	0.988
Basciani et al. (II) [33]	0.497	[0.038, 0956]	0.0	2.77	0.973
Basciani et al. (I and II) [33]	0.536	[0.066, 1.006]	0.0	2.18	0.975
Hill et al. (I) [38]	0.499	[0.022, 0.977]	0.0	2.78	0.972
Hill et al. (II) [38]	0.508	[0.032, 0.985]	0.0	2.78	0.972
Hill et al. (I and II) [38]	0.507	[−0.005, 1.018]	0.0	2.78	0.948
Kjølbæk et al. (I) [40]	0.557	[0.087, 1.026]	0.0	2.18	0.988
Kjølbæk et al. (II) [40]	0.522	[0.049, 0.996]	0.0	2.71	0.975
Kjølbæk et al. (I and II) [40]	0.586	[0.088, 1.084]	0.0	2.05	0.979
Lynch et al. [41]	0.465	[−0.024, 0.954]	0.0	2.65	0.977
Mobley et al. (I) [43]	0.411	[−0.066, 0.888]	0.0	1.56	0.997
Mobley et al. (II) [43]	0.543	[0.065, 1.021]	0.0	2.54	0.980
Mobley et al. (I and II) [43]	0.445	[−0.067, 0.956]	0.0	1.43	0.994
Vupadhyayula et al. (I) [49]	0.488	[0.030, 0.945]	0.0	2.68	0.976
Vupadhyayula et al. (II) [49]	0.485	[0.019, 0.952]	0.0	2.72	0.974
Vupadhyayula et al. (I and II) [49]	0.470	[−0.006, 0.945]	0.0	2.61	0.956

Table S4. Sensitivity analysis for strength outcomes following the removal of single groups or randomized controlled trials to assess the robustness of meta-analyses results.

Group excluded	Mean difference	[95% CI]	I^2 (%)	χ^2	P for χ^2
**1-RM squat (kg)**					
None	−0.938	[−4.571, 2.696]	0.0	0.81	0.667
Banaszek et al. [32]	−0.533	[−4.937, 3.871]	0.0	0.71	0.400
DeNysschen et al. [35]	−2.523	[−7.791, 2.746]	0.0	0.15	0.710
Volek et al. [48]	−0.371	[−4.326, 3.585]	0.0	0.31	0.580
**Grip strength (kg)**					
None	−0.493	[−1.282, 0.296]	0.0	2.24	0.692
Basciani et al. (I) [33]	−0.649	[−1.476, 0.178]	0.0	0.71	0.871
Basciani et al. (II) [33]	−0.570	[−1.389, 0.249]	0.0	1.76	0.623
Basciani et al. (I and II) [33]	−0.747	[−1.608, 0.115]	0.0	0.07	0.966
Thomson et al. [46]	−0.458	[−1.368, 0.452]	0.0	2.21	0.529
Vupadhyayula et al. (I) [49]	−0.332	[−1.268, 0.604]	0.0	1.84	0.606
Vupadhyayula et al. (II) [49]	−0.389	[−1.336, 0.557]	0.0	2.09	0.555
Vupadhyayula et al. (I and II) [49]	−0.035	[−1.272, 1.202]	0.0	1.32	0.516
**Leg/knee extension (Nm)**					
None	−3.012	[−19.253, 13.229]	85.8	21.12	0.000
Haub et al. [37]	4.720	[−8.320, 17.760]	62.1	5.28	0.072
Lynch et al. [41]	−9.000	[−28.443, 10.443]	83.5	12.10	0.002

Thomson et al. [46]	−7.698	[−30.935, 15.539]	88.9	18.07	0.000
Tomayko et al. [47]	0.952	[−16.399, 18.304]	89.2	18.46	0.000
**Leg/knee flexion (Nm)**					
None	2.926	[−1.705, 7.557]	0.0	0.53	0.768
Haub et al. [37]	3.418	[−1.771, 8.606]	0.0	0.36	0.549
Lynch et al. [41]	0.396	[−8.087, 8.880]	0.0	0.04	0.838
Tomayko et al. [47]	3.326	[−1.541, 8.193]	0.0	0.25	0.614

Study	Animal protein			Plant protein			Weight	Mean difference
	Sample size	Mean (kg)	SD (kg)	Sample size	Mean (kg)	SD (kg)	(%)	(kg) [95% CI]
**Without RET**								
Basciani (I) [33]	16	−3.02	2.61	16	−2.62	2.77	1.52	−0.40 [−2.26, 1.46]
Basciani (II) [33]	16	−0.65	4.10	16	−2.62	2.77	0.93	1.97 [−0.46, 4.40]
Hill (I) [38]	21	−1.48	2.38	21	−1.45	2.35	2.44	−0.03 [−1.46, 1.40]
Hill (II) [38]	20	−1.04	2.44	21	−1.45	2.35	2.33	0.41 [−1.06, 1.88]
Kjølbæk (I) [40]	38	1.87	1.70	36	1.58	1.43	7.14	0.29 [−0.42, 1.00]
Kjølbæk (II) [40]	39	1.94	1.34	36	1.58	1.43	8.20	0.36 [−0.27, 0.99]
Moeller (I) [44]	21	0.4	1.2	24	0.4	1.1	7.65	0.00 [−0.67, 0.67]
Moeller (II) [44]	21	0.4	1.2	24	0.8	0.9	8.20	−0.40 [−1.03, 0.23]
Neacsu [45]	20	−0.65	1.43	20	−0.67	0.94	6.67	0.02 [−0.72, 0.76]
Tomayko [47]	11	0.4	3.3	12	−2.2	4.9	0.49	2.60 [−0.79, 5.99]
Vupadhyayula (I) [49]	35	0.05	1.53	39	−0.08	4.65	2.13	0.13 [−1.42, 1.68]
Vupadhyayula (II) [49]	35	0.05	1.53	36	−0.18	1.45	7.39	0.23 [−0.46, 0.92]
Subtotal ($\tau^2 = 0.000$; $\chi^2 = 8.49$; df = 11 [$p = 0.669$]; $I^2 = 0.0\%$)							**55.07**	**0.11 [−0.15, 0.37]**
**With RET**								
Hartman [36]	18	3.97	1.20	19	2.85	1.89	4.27	1.12 [0.10, 2.14]
Haub [37]	10	−0.2	1.7	11	−0.9	1.3	2.83	0.70 [−0.61, 2.01]
Joy [39]	12	3.2	1.4	12	2.5	1.5	3.50	0.70 [−0.46, 1.86]
Lynch [41]	26	1.5	0.3	22	1.2	0.3	16.95	0.30 [0.12, 0.48]
Mobley (I) [43]	17	2.26	2.33	15	3.23	2.48	1.87	−0.97 [−2.64, 0.70]
Mobley (II) [43]	14	2.13	2.52	15	3.23	2.48	1.58	−1.10 [−2.92, 0.72]
Thomson [46]	34	1.0	1.0	26	1.4	1.2	9.11	−0.40 [−0.97, 0.17]
Volek [48]	19	3.3	1.5	22	1.8	1.6	4.82	1.50 [0.56, 2.44]
Subtotal ($\tau^2 = 0.2634$; $\chi^2 = 19.86$; df = 7 [$p = 0.006$]; $I^2 = 64.8\%$)							**44.93**	**0.34 [−0.16, 0.84]**
**Overall** ($\tau^2 = 0.0803$; $\chi^2 = 29.71$; df = 19 [$p = 0.056$]; $I^2 = 36.1\%$)							**100.00**	**0.22 [−0.02, 0.46]**

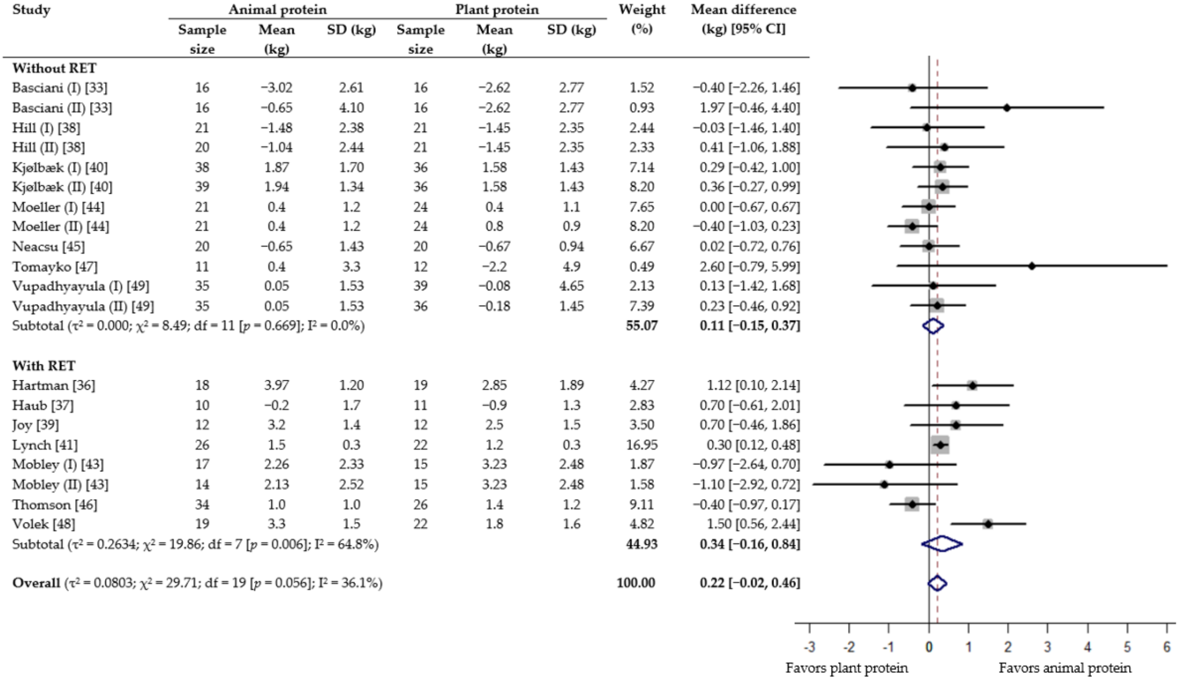

Figure S1. Effect of consuming animal protein compared to plant protein on changes in absolute lean mass (kg), with and without resistance exercise training (RET). Data expressed as weighted mean differences with 95% CIs, using a random-effects model.

Study	Animal protein			Plant protein			Weight	Mean difference
	Sample size	Mean (%)	SD (%)	Sample size	Mean (%)	SD (%)	(%)	(%) [95% CI]
**Without RET**								
Basciani (I) [33]	16	2.12	3.40	16	2.45	2.77	4.34	−0.33 [−2.49, 1.83]
Basciani (II) [33]	16	3.06	3.36	16	2.45	2.77	4.42	0.61 [−1.53, 2.75]
Hill (I) [38]	21	1.77	2.48	21	1.25	1.84	11.69	0.52 [−0.79, 1.83]
Hill (II) [38]	20	1.70	2.44	21	1.25	1.84	11.35	0.45 [−0.88, 1.78]
Kjølbæk (I) [40]	38	0.58	3.66	36	0.66	3.01	8.62	−0.08 [−1.61, 1.45]
Kjølbæk (II) [40]	39	0.98	3.21	36	0.66	3.01	10.12	0.32 [−1.09, 1.73]
Vupadhyayula (I) [49]	35	0.81	4.10	39	−0.07	6.23	3.58	0.88 [−1.49, 3.25]
Vupadhyayula (II) [49]	35	0.81	4.10	36	0.10	3.00	7.26	0.71 [−0.96, 2.38]
Subtotal ($\tau^2 = 0.000$; $\chi^2 = 1.19$; df = 7 [$p = 0.991$]; $I^2 = 0.0\%$)							**61.37**	**0.38 [−0.19, 0.95]**
**With RET**								
Lynch [41]	26	1.9	2.4	22	1.2	1.6	15.60	0.70 [−0.44, 1.84]
Mobley (I) [43]	17	1.44	1.71	15	0.23	2.09	11.35	1.21 [−0.12, 2.54]
Mobley (II) [43]	14	0.42	1.49	15	0.23	2.09	11.69	0.19 [−1.12, 1.50]
Subtotal ($\tau^2 = 0.000$; $\chi^2 = 1.14$; df = 2 [$p = 0.565$]; $I^2 = 0.0\%$)							**38.63**	**0.70 [−0.03, 1.42]**
**Overall** ($\tau^2 = 0.000$; $\chi^2 = 2.78$; df = 10 [$p = 0.986$]; $I^2 = 0.0\%$)							**100.00**	**0.50 [0.05, 0.95]**

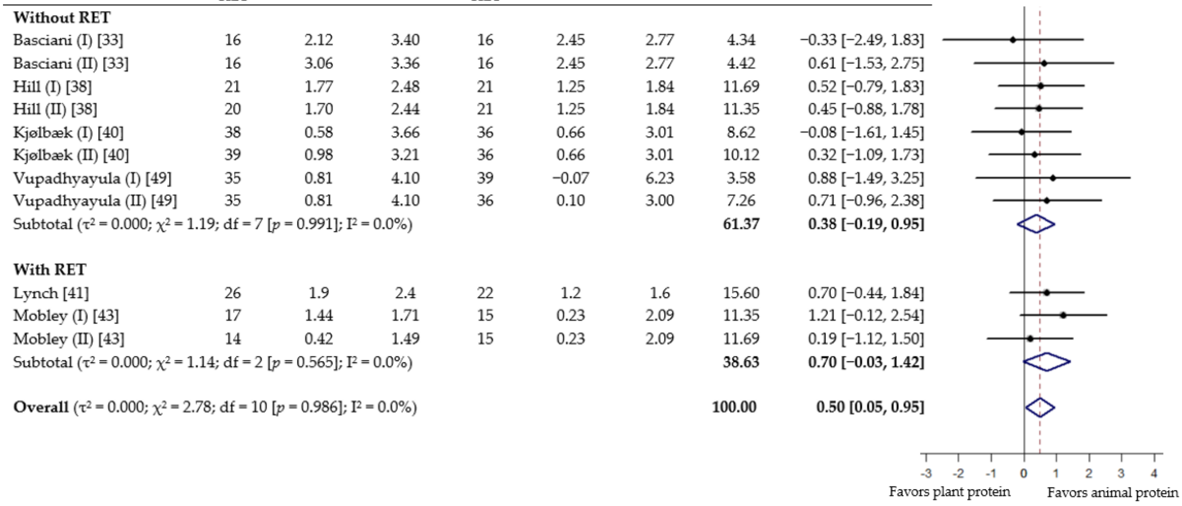

Figure S2. Effect of consuming animal protein compared to plant protein on changes in percent lean mass (%), with and without resistance exercise training (RET). Data expressed as weighted mean differences with 95% CIs, using a random-effects model.

Study	Animal protein			Plant protein			Weight (%)	Mean difference (kg) [95% CI]
	Sample size	Mean (kg)	SD (kg)	Sample size	Mean (kg)	SD (kg)		
**Without RET**								
Basciani (I) [33]	16	1.98	3.97	16	0.88	3.65	8.90	1.10 (−1.15, 3.75)
Basciani (II) [33]	16	1.39	4.80	16	0.88	3.65	7.11	0.51 (−2.45, 3.47)
Vupadhyayula (I) [49]	52	−1.59	4.27	57	−0.70	3.51	28.83	−0.89 (−2.36, 0.58)
Vupadhyayula (II) [49]	52	−1.59	4.27	48	−0.86	2.92	30.43	−0.73 (−2.16, 0.70)
Subtotal (τ^2 = 0.0000; χ^2 = 2.21; df = 3 [p = 0.529]; I^2 = 0.0%)							**75.28**	**−0.46 [−1.37, 0.45]**
**With RET**								
Thomson [46]	34	1.0	3.1	26	1.6	3.1	24.72	−0.60 (−2.19, 0.99)
Subtotal							**24.72**	**−0.60 [−2.19, 0.99]**
**Overall** (τ^2 = 0.000; χ^2 = 2.24; df = 4 [p = 0.692]; I^2 = 0.0%)							**100.00**	**−0.49 [−1.28, 0.30]**

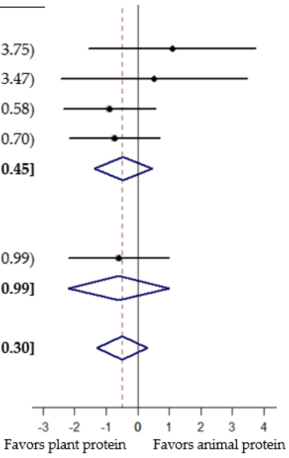

Figure S3. Effect of consuming animal protein compared to plant protein on changes in grip strength (kg), with and without resistance exercise training (RET). Data expressed as weighted mean differences with 95% CIs, using a random-effects model.

Study	Animal protein			Plant protein			Weight (%)	Mean difference (Nm) [95% CI]
	Sample size	Mean (Nm)	SD (Nm)	Sample size	Mean (Nm)	SD (Nm)		
**With RET**								
Haub [37]	10	27	11	11	43	11	28.67	−16.00 [−25.43, −6.57]
Lynch [41]	26	40	17	22	28	18	28.36	12.00 [2.04, 21.96]
Thomson [46]	34	25.5	22.1	26	18.4	18.6	28.14	7.10 [−3.21, 17.41]
Subtotal (τ^2 = 209.6009; χ^2 = 18.46; df = 2 [p = 0.000]; I^2 = 89.2%)							**85.17**	**0.95 [−16.40, 18.30]**
**Without RET**								
Tomayko [47]	11	−12.9	48.8	12	12.9	19.1	14.83	−25.80 [−56.59, 4.99]
Subtotal							**14.83**	**−25.80 [−56.59, 4.99]**
**Overall** (τ^2 = 216.3225; χ^2 = 21.12; df = 3 [p = 0.000]; I^2 = 85.8%)							**100.00**	**−3.01 [−19.25, 13.23]**

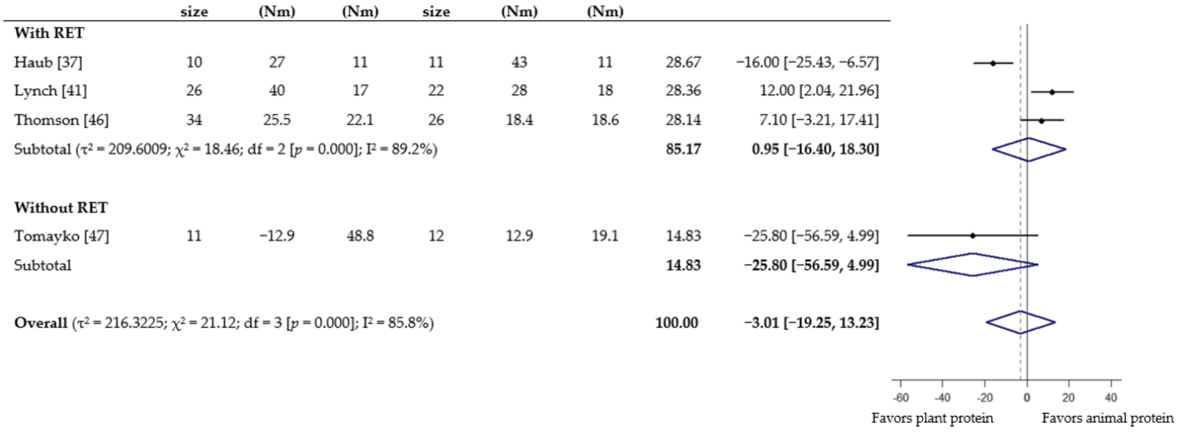

Figure S4. Effect of consuming animal protein compared to plant protein on changes in peak torque of leg/knee extension (Nm), with and without resistance exercise training (RET). Data expressed as weighted mean differences with 95% CIs, using a random-effects model. Nm: newton meter.

Study	Animal protein			Plant protein			Weight	Mean difference
	Sample size	Mean (Nm)	SD (Nm)	Sample size	Mean (Nm)	SD (Nm)	(%)	(Nm) [95% CI]
**With RET**								
Haub [37]	10	30	12	11	29	12	20.33	1.00 [−9.27, 11.27]
Lynch [41]	26	20	8	22	16	11	70.20	4.00 [−1.53, 9.53]
Subtotal (τ^2 = 0.0000; χ^2 = 0.25; df = 1 [p = 0.614]; I^2 = 0.0%)							**90.53**	**3.33 [−1.54, 8.19]**
**Without RET**								
Tomayko [47]	11	2.4	14.3	12	3.3	22.0	9.47	−0.90 [−15.95, 14.15]
Subtotal							**9.47**	**−0.90 [−15.95, 14.15]**
**Overall** (τ^2 = 0.000; χ^2 = 0.53; df = 2 [p = 0.768]; I^2 = 0.0%)							**100.00**	**2.93 [−1.70, 7.56]**

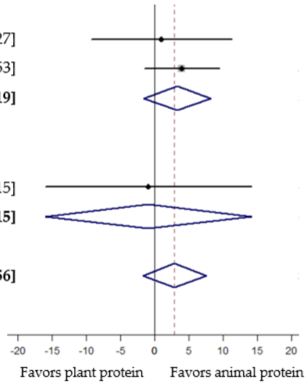

Figure S5. Effect of consuming animal protein compared to plant protein on changes in peak torque of leg/knee flexion (Nm), with and without resistance exercise training (RET). Data expressed as weighted mean differences with 95% CIs, using a random-effects model. Nm: newton meter.
